# Phenolphthalein Anilide Based Poly(Ether Sulfone) Block Copolymers Containing Quaternary Ammonium and Imidazolium Cations: Anion Exchange Membrane Materials for Microbial Fuel Cell

**DOI:** 10.3390/membranes11060454

**Published:** 2021-06-20

**Authors:** Aruna Kumar Mohanty, Young Eun Song, Jung Rae Kim, Nowon Kim, Hyun-jong Paik

**Affiliations:** 1 Department of Polymer Science and Engineering, Pusan National University, Busan 46241, Korea; akmohanty07@pusan.ac.kr; 2 School of Chemical and Biomolecular Engineering, Pusan National University, Busan 46241, Korea; duddms37@gmail.com (Y.E.S.); j.kim@pusan.ac.kr (J.R.K.); 3 Department of Environmental Engineering, Dong-eui University, Busan 47340, Korea

**Keywords:** cardo group, anion exchange membrane, quaternary ammonium, imidazolium, hydroxide conductivity, microbial fuel cell

## Abstract

A class of phenolphthalein anilide (PA)-based poly(ether sulfone) multiblock copolymers containing pendant quaternary ammonium (QA) and imidazolium (IM) groups were synthesized and evaluated as anion exchange membrane (AEM) materials. The AEMs were flexible and mechanically strong with good thermal stability. The ionomeric multiblock copolymer AEMs exhibited well-defined hydrophobic/hydrophilic phase-separated morphology in small-angle X-ray scattering and atomic force microscopy. The distinct nanophase separated membrane morphology in the AEMs resulted in higher conductivity (IECw = 1.3–1.5 mequiv./g, σ(OH^−^) = 30–38 mS/cm at 20 °C), lower water uptake and swelling. Finally, the membranes were compared in terms of microbial fuel cell performances with the commercial cation and anion exchange membranes. The membranes showed a maximum power density of ~310 mW/m^2^ (at 0.82 A/m^2^); 1.7 and 2.8 times higher than the Nafion 117 and FAB-PK-130 membranes, respectively. These results demonstrated that the synthesized AEMs were superior to Nafion 117 and FAB-PK-130 membranes.

## 1. Introduction

Anion exchange membranes (AEMs) are generally applied in various electrochemical devices such as fuel cells, capacitors (in deionization), flow batteries, and electrolyzers. In microbial fuel cells (MFCs), ion exchange membranes are employed to physically separate the anodic and cathodic chambers and to provide pathways for the ions transport. MFCs are a promising energy recovery platform because the electricity is generated from wastewater (or biologically degradable organic materials) using enriched electro-active bacteria [1]. MFCs have advantages over typical polymer electrolyte fuel cells (PEFCs) because electrons at the anode are generated by microbes assisted metabolic oxidation processes in contrast to the expensive noble metal catalyst mediated oxidation. The power generations in MFCs mainly depend on two factors: (1) ion-exchange membrane performance and (2) reduction kinetics at the cathode [2,3]. Due to the higher transport of cations other than protons through cation exchange membrane (CEM) (e.g., Nafion) which usually leads to pH splitting into the chambers, AEMs are seen as an alternative for use in MFC. In pH splitting, lower pH in the anode chamber affects the microbial activity while the higher pH in the cathode chamber reduces the cathode potential [2]. Instead, AEMs in MFC can allow a higher rate of proton transfer in the form of buffered anions for better performance [2,4]. Although AEMs are promising, there is not enough research on AEMs as separators in MFCs.

AEMs are expected to possess higher ion conductivity with the combination of good mechanical strength, higher chemical, and thermal stability. Over the years, a variety of AEMs was prepared especially based on poly(arylene ethers) with functional groups such as quaternized ammonium [5], imidazolium [6], phosphonium [7], guanidinium [8], morpholinium [9], piperidinium [10] due to their good solubility in solvents, higher thermal and mechanical properties. The AEMs are generally prepared via chloromethylation or bromination of benzylmethyl groups followed by quaternization (Menshutkin) and hydroxide exchange reactions. Unlike CEMs, the AEMs are, however, generally reported with a wide range of lower ion conductivity. The poor ion conductivity in AEMs is due to the inherently lower OH-ion mobility (OH-conductivity, 1.76 times lesser than H+ in dilute solution [11]) and relatively poor phase separated morphology. Generally, very high IECw of membranes results in excessive water uptake (swelling) leading to poor mechanical strength and poorer ion conductivity (ion dilution). In contrast, studies have shown that ionomeric membranes at a lower IECw can show higher ion conductivity due to well-defined phase separated morphology. For example, in our previously synthesized membranes [12], controlled ordering of the sequenced hydrophilic and hydrophobic blocks in multiblock copolymers led to distinct well-defined hydrophilic/hydrophobic phase-separated morphology which resulted in higher ion conductivity, lower uptake and swelling. However, there is scope to further improve the ion conductivity of the AEMs without affecting the membrane properties.

In ionomeric membranes, hydrophilic domains are responsible for ion conductivity while hydrophobic domains provide mechanical strength and thermal stability. Thus, there has always been a challenge to optimize the mechanical, chemical, and thermal stability with the ion conductivity of a membrane at an optimum IECw for actual applications. It has been known that cardo groups (e.g., fluorene, phenolphthalein, etc.) in polymers improve thermal and mechanical properties due to their bulky and fully aromatic structure [13]. Thus, researchers have reported AEMs based on cardo groups in recent years. Miyatake et al. [14] reported fluorene-based random and multiblock copolymer AEMs using the chloromethylation route with high ion conductivity (random AEM, rQPE, 35 mS/cm at 60 °C and multiblock AEM, QPE-X22Y23, 126 mS/cm at 60 °C) and thermal stability. AEMs were also reported on phenolphthalein-based poly(ether sulfone) [15] and poly(ether ketone) [16] polymers (using chloromethylation route). The chloromethylation route uses carcinogenic reagents like chloromethyl methyl ether and has lots of disadvantages such as poor control over the degree of functionalization, sites of functionalization in the polymer, and probable side reactions such as crosslinking while intending for a higher degree of functionalization [17]. In an alternative method, Zhang et al. reported a class of poly(ether sulfone) AEMs using precursor tertiary amine-functionalized phenolphthalein monomers for the later Menshutkin reaction with methyl iodide [18]. In the report, the quaternary ammonium group on the flexible side chain instead of the main chain facilitated the ion aggregation in the form of ion clusters. However, the low ion group density per unit of the random copolymer, and the small ion clusters in the absence of a network structure among hydrophilic domains led to a lower ion conductivity in the membrane. In a slightly different technique, Rao et al. [19] used benzylmethyl bromination to synthesize imidazolium functionalized phenolphthalein-based poly(ether sulfone) AEMs which exhibited high thermal stability and ion conductivity (PI-PES, ~100 mS/cm at 80 °C). The cardo groups in the polymer structure provided a large free volume for water molecules which helped the membrane with better ion conduction without much water swelling [18,19].

**Scheme 1:**
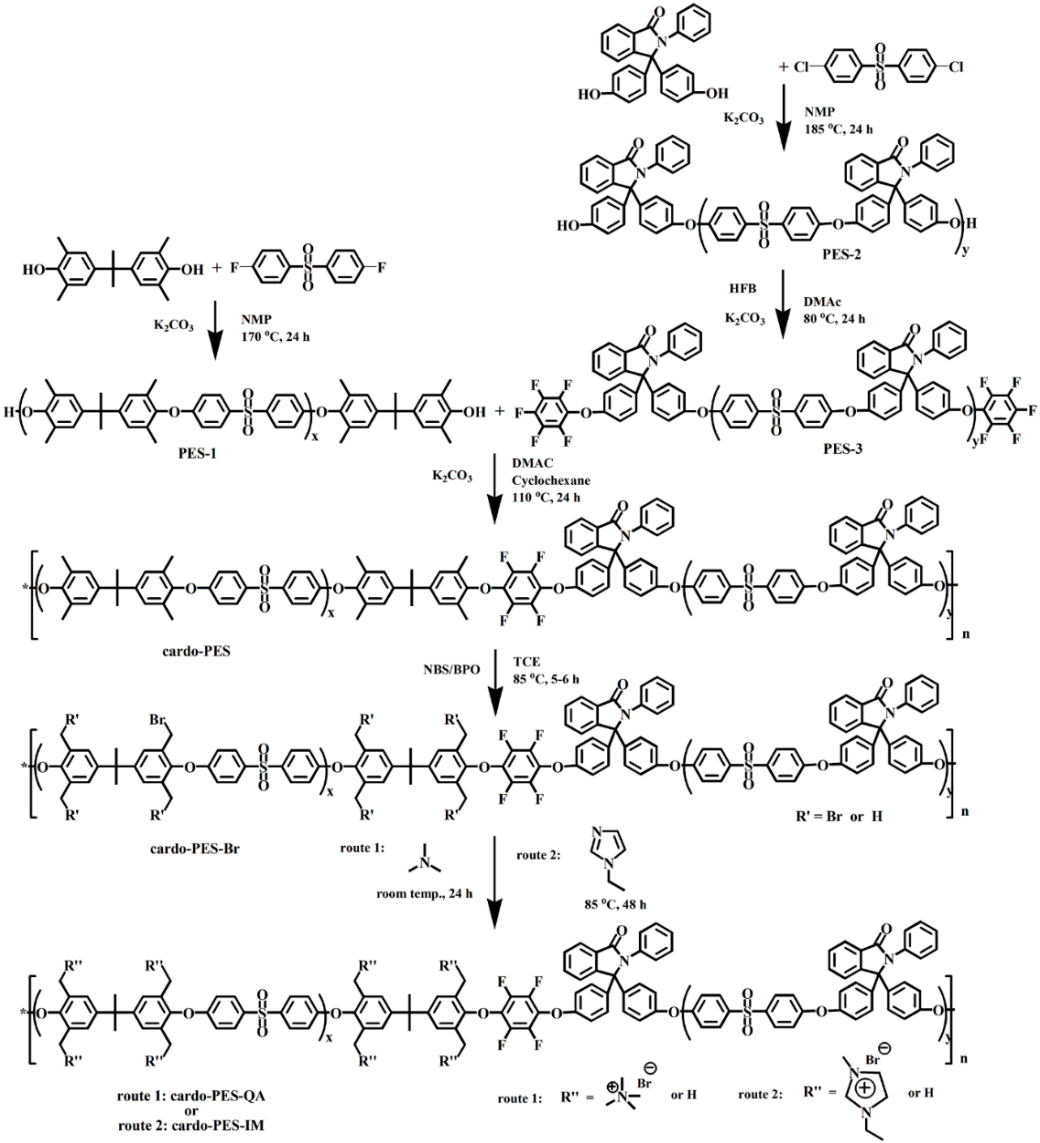
Synthesis of the anion exchange cardo-PES multiblock copolymers.

Here, we report the synthesis and properties of quaternary ammonium- and imidazolium-functionalized N–phenyl–3,3– bis(4–hydroxyphenyl)phthalimidine (or phenolphthalein anilide, PA)-based multiblock poly(ether sulfone) (PES) copolymers as novel AEM materials (Scheme 1). The AEMs are synthesized by a low-temperature polycondensation (using HFB as a linkage group) reaction between telechelic oligomer blocks to synthesize a precursor multiblock copolymer followed by benzylmethyl bromination and quaternization. The low-temperature reaction of HFB prevents the randomization of blocks by trans-etherification [20]. The 2,2–bis(4–hydroxy–3,5–dimethylphenyl) propane (tBPA) is used in the precursor hydrophilic block for bromomethylation by N–bromosuccinimide (NBS) and subsequent quaternization. Meanwhile, PA is used in the hydrophobic block for good polymer solubility, higher dimensional and thermal stability. The block structure of the copolymer can provide a well-defined phase-separated morphology for higher conductivity. Overall, membrane properties including water uptake and swelling properties are evaluated. Besides, the ionomeric membranes are tested in a dual-chambered microbial fuel cell (MFC) and performances are compared with the state-of-the-art CEM, Nafion 117 (Dupont, Wilmington, DE, USA), and an AEM, FAB-PK-130 (Fumasep, Fumatech BWT GmbH, Germany).

## 2. Experimental

### 2.1. Materials

Hexafluoro benzene (HFB, 99%), 4,4′–difluorodiphenyl sulfone (DFDPS, +98%), and 4,4′–dichlorodiphenyl sulfone (DCDPS, 99%), N–bromosuccinimide (NBS, 99%) were purchased from Alfa Aesar and used without further purification. 2,2–Bis(4–hydroxy–3,5–dimethylphenyl)propane (tBPA, +98%) was obtained from TCI and used as received. N-phenyl–3,3–bis(4–hydroxyphenyl)phthalimidine (PA) was prepared according to the procedure reported in the literature [13,21]. Anhydrous potassium carbonate (K_2_CO_3_, MerckKenilworth, NJ, USA) was dried at 100 °C for 10 h under a vacuum. NMP and DMAc were purchased from Daejung Chemicals and were purified by distillation over P_2_O_5_ under reduced pressure. Toluene (Daejung Chemicals, Siheung-si, Korea) was freshly distilled over Na before use. Benzoyl peroxide (BPO, 70–90%) was purchased from Sigma Aldrich (St. Louis, MO, USA) and used as received. 1,1,2,2–Tetrachloroethane (TCE) was obtained from Daejung Chemicals (Siheung-si, Korea) and used as received. The Nafion 117 (Dupont, Wilmington, DE, USA), and FAB-PK-130 (Fumasep, Fumatech BWT GmbH, Bietigheim-Bissingen, Germany) membranes, solvents, and other chemicals were used as received.

### 2.2. Synthesis of the –OH Terminated Telechelic Block, PES-1 

The PES-1 was synthesized as follows: DFDPS (4.50 g, 17.7 mmol), tBPA (5.70 g, 20 mmol), K_2_CO_3_ (6.9 g, 50.1 mmol), NMP (70 mL), and toluene (40 mL) were charged into a 250 mL three-neck round bottom flask equipped with a magnetic stirrer, N_2_ inlet, and Dean-Stark trap fitted with a condenser with a CaCl_2_ filter. The solution was heated at 150 °C (~4 h) for azeotrope distillation of toluene and water. Then, the temperature was raised to 170 °C for 20 h. After cooling down to room temperature, the viscous solution was poured slowly into a stirring acidified methanol in a beaker. The powder product was isolated as an off-white powder after washing with methanol and water, filtration, and drying under vacuum (yield: 92%). The synthesized oligomer product was characterized by ^1^H NMR spectroscopy. The molecular weight was determined as Mn~5.1 kDa in SEC.

### 2.3. Synthesis of the F-Terminated Telechelic Block, PES-3 

For the synthesis of PES-3, a -OH terminated telechelic block, PES-2 was first synthesized as follows: DCDPS (8.76 g, 30.5 mmol), PA (12.95 g, 32.9 mmol), K_2_CO_3_ (11.3 g, 82.2 mmol), NMP (180 mL), toluene (75 mL) in a 250 mL three-neck flask were heated as explained for PES-1 (160 °C for 4 h and then at 185 °C for 24 h). Then, the oligomer was isolated as the off-white powder after precipitation in methanol, washing, and drying in a vacuum (yield: 94%). The synthesized oligomer was characterized by ^1^H NMR spectroscopy. The molecular weight was determined as Mn~8.1 kDa in SEC. 

The fluorine end-capping of the PES-2 was carried out as follows: PES-2 (11 g, 1.38 mmol), K_2_CO_3_ (0.57 g, 4 mmol), DMAc (110 mL), and cyclohexane (50 mL) were added to a three-necked 250 mL flask equipped with a magnetic stirrer, N_2_ inlet, and Dean-Stark trap fitted with the condenser with a CaCl_2_ filter. The solution was refluxed at 110 °C for 2–3 h to remove the water with cyclohexane in an azeotrope process. The solution was cooled down to 80 °C. After stopping the nitrogen flow, excess HFB (2.55 g, 13.7 mmol) was added to the flask and the solution was stirred overnight. Finally, the product was isolated as an off-white powder after precipitation in methanol, washing, filtration, and drying (yield: 95%). The oligomer was characterized by ^1^H NMR and ^19^F NMR spectroscopy. The molecular weight of PES-3 was determined as Mn~8.5 kDa in SEC.

### 2.4. Synthesis of the Precursor Multiblock Copolymer, Cardo-PES

The multiblock copolymer, cardo-PES was synthesized by a low-temperature coupling reaction between the PES-1 and the PES-3 as mentioned in the HFB end-capping reaction. The procedure was as follows: The solution mixture of PES-1 (Mn (NMR) = 5.1 kDa) (9 g, 1.75 mmol), K_2_CO_3_ (0.96 g, 6.9 mmol), DMAc (200 mL), and cyclohexane (70 mL) in a three-necked 500 mL flask was heated at 110 °C for 2–3 h to remove the water in the system by forming an azeotrope with cyclohexane. Then, PES-3 (Mn (NMR) = 8.6 kDa) (15.2 g, 1.75 mmol) was added to the flask at room temperature and again heated to 105–110 °C for 48 h. Finally, the multiblock copolymer was isolated as a fibrous product after precipitation in acidified methanol solution, washing and drying at 120 °C under vacuum for 24 h (yield: 95%). The polymer product was characterized by ^1^H NMR and ^19^F NMR spectroscopy. The molecular weight of the cardo-PES was determined as Mn = 27 kDa in SEC.

### 2.5. Synthesis of the Benzyl Brominated Multiblock Copolymer, PES-Br

The bromination of benzylmethyl groups in the cardo-PES was carried out using NBS/BPO. The procedure was as follows: cardo-PES (15.5 g) was dissolved in TCE (310 mL) in a 500 mL three-necked round-bottomed flask equipped with a magnetic stirrer, and a condenser with a nitrogen balloon. Then, the flask was added NBS (8.23 g, 46 mmol) and BPO (0.56 g, 2.3 mmol) and heated to 85 °C for 5 h. After cooling to room temperature, the solution was precipitated in ethanol followed by multiple washing and drying (at 60 °C) under vacuum to afford a light-yellow solid (90% yield). The brominated polymer product was characterized by ^1^H NMR spectroscopy.

### 2.6. Synthesis of Cardo-PES-QA and Cardo-PES-IM

The cardo-PES-Br was reacted with trimethylamine solution (50 wt% in water) (at room temperature for 24 h) and ethyl imidazole (at 85 °C for 24 h) to obtain the corresponding ammonium (QA) and imidazolium (IM) functionalized multiblock copolymers. As a typical example, the cardo-PES-IM was synthesized as follows: ethyl imidazole (3.4 g) was added dropwise to the solution of cardo-PES-Br (4 g) in DMF (35 mL) and heated to 85 °C for 24 h under nitrogen. The cardo-PES-IM was isolated as a brown powder after precipitating in ethyl acetate, washing, and drying at 65 °C under vacuum. 

### 2.7. Fabrication of Membranes

The cardo-PES-QA and cardo-PES-IM membranes were prepared by solution-casting using the DMF solutions. The cardo-PES-QA or cardo-PES-IM (1.1 g) polymer was first dissolved in DMF (20.0 mL) by stirring at room temperature. Then, the solution was filtered through a plug of cotton onto a Petri dish and heated at 55 °C in an oven for 12 h. After the evaporation of the solvent, the membrane was peeled off by immersing in deionized water. The transparent and flexible membranes were obtained with thicknesses in the range of 100–150 µm. The membranes were converted to the OH^−^, HCO_3_^−^, and Cl^−^ form by immersing them in 1 M NaOH, 1 M NaHCO_3_, and 1 M NaCl solutions for 24 h followed by thorough washing. The resultant membranes were stored in deionized water for future tests.

### 2.8. Characterization

^1^H NMR experiments were carried out using a Varian Unity Inova 500 spectrometer (400 MHz) or 500 MHz Agilent Superconducting FT-NMR Spectrometer using CDCl_3_ or DMSO-d_6_. The peak signals were identified in comparison to the solvent residue peaks (CDCl_3_, at 7.26 ppm and DMSO-d_6_, at 2.54 ppm). The infrared spectra were recorded in transmittance mode on a Nicole 6700 FT-IR spectrophotometer. Thermogravimetric analysis (TGA) was done using TGA Q5000 (TA Instruments, New Castle, DE, USA) at a heating rate of 10 °C/min under N2. The membrane samples were preheated at 100 °C for at least 12 h under a vacuum before the experiment. X-ray photoelectron spectra (XPS) data were obtained using an AR-XPS system (Theta probe, Thermo Scientific, Waltham, MA, USA). Atomic force microscopy (AFM) images were taken in tapping mode with n-Tracer SPM (Nanofocus), equipped with a silicon cantilever of curvature of <8 nm and resonance frequency of ~320 kHz. The scanning electron microscopy (SEM) images were obtained using Philips XL 30 FEG microscope (Philips, Hillsboro, OR, USA) with an accelerating voltage of 10 kV. The synchrotron small-angle X-ray scattering (SAXS) experiments were carried out using the BL4C beamline at the Pohang Accelerator Laboratory (PAL) to investigate the nature of ion clusters in the AEMs. Data was collected in the *q*-range 0.1–1.8 nm-1 using a wavelength (λ) of the incident X-ray beam at 0.07 nm and Rayonix 2D SX 165 detector(Rayonix, Evanston, IL, USA). Pre-equilibrated membranes (Br- form) at RH ~75% mounted on the window of a brass holder were measured at the condition of ambient temperature under vacuum. The scattering intensities were normalized for background scattering with an empty sample holder. A constant sample-to-detector distance was maintained between 5 to 0.2 m. Scattering vectors (*q*) were calculated from q = 4πsin*θ/λ*, where *θ* is the scattering angle between the incident and scattered radiation, and *λ* is the wavelength of the X-ray beam source. The characteristic separation length (d) was calculated using Bragg’s law: d = 2π/q. 

The water uptake and swelling ratio were calculated from the ratio of the changes in the weight and the length in the wet state to the dry state of membranes (in OH^−^ form). After drying at 90 °C under vacuum for 24 h, the membranes were immersed in water for at least 24 h. Then, the swelled membranes were taken out of the water, quickly wiped the excess surface water with blotting paper and measured the weight and length. Repeated measurements were taken to obtain a constant value of weight and length. The water uptake (WUw) was calculated from:

(1) $${\mathrm{WU}}_{w}\left(\%\right)=\left(\frac{W_{\mathrm{wet}}-W_{\mathrm{dry}}}{W_{\mathrm{dry}}}\right)\times 100$$

where W_wet_ and W_dry_ are the weights of the membrane in wet and dry states, respectively.

The swelling ratio was calculated from:

(2) $$\Delta l\left(\%\right)=\left(\frac{l_{\mathrm{wet}}-l_{\mathrm{dry}}}{l_{\mathrm{dry}}}\right)\times 100$$

(3) $$\Delta t\left(\%\right)=\left(\frac{t_{\mathrm{wet}}-t_{\mathrm{dry}}}{t_{\mathrm{dry}}}\right)\times 100$$

where l_wet_ and l_dry_ are the lengths of the membrane in wet and dry states, respectively.

The hydration number (λ) representing the average number of water molecules per ionic group was calculated from:

(4) $$\lambda =\frac{{\mathrm{WU}}_{W}\left(\%\right)\times 10}{{\mathrm{IEC}}_{W}\times {\;M}_{W,H2O}}$$

where M_W,H2O_ is the molecular mass of water (18 g/mol). IECw is the weight-based ion exchange capacity.

The IECw of the membrane (Br^−^ form) was determined by Mohr’s method as the moles of ionic groups per gram of the dry polymer (mmole/g or mequiv./g). The dried membrane of ca. 0.1 g was immersed in a 50 mL of 0.2 M NaNO_3_ solution for 48 h to allow the release of Br^-^ ions into the solution in exchange with the NO_3_^-^ ions. The liberated Br^-^ in the solution was titrated with a 0.01 M AgNO_3_ solution using K_2_CrO_4_ as a colorimetric indicator. The IECw was calculated from:

(5) $${\mathrm{IEC}}_{W,\;\mathrm{Titr}.\;}=\frac{C_{\mathrm{AgNO}3}\times {\;\Delta V}_{\mathrm{AgNO}3}}{W_{dry}}$$

where Wdry is the weight of the dry membrane, ΔV_AgNO3_ is the consumed amount of AgNO_3_ solution, and C_AgNO3_ is the concentration of AgNO_3_ solution.

The in-plane ion conductivity of the membrane (in OH^−^, Cl^−^, and HCO_3_^−^ form) was calculated by measuring the resistance (R) in a two-probe method using electrochemical impedance spectroscopy (Solatron, potentiostat). The experiments are carried out over a frequency range of 5 Hz–1 MHz with a sinusoidal perturbation amplitude of 10 mV. The measurements were taken on fully hydrated membranes with the homemade conductivity cell immersed in the degassed deionized water over a temperature range. The ionic conductivity (σ, S/cm) was calculated from:

(6) $$\sigma =\frac{l}{R\times A}$$

where *l* is the distance between the platinum electrodes in the conductivity cell, cross-sectional area, A is calculated from the thickness (t) and width (w) of the membrane, and resistance, R (Ω) is derived from the intercept of the low-frequency complex impedance with the Re(*Z*) axis.

### 2.9. Microbial Fuel Cell 

**Figure 1:**
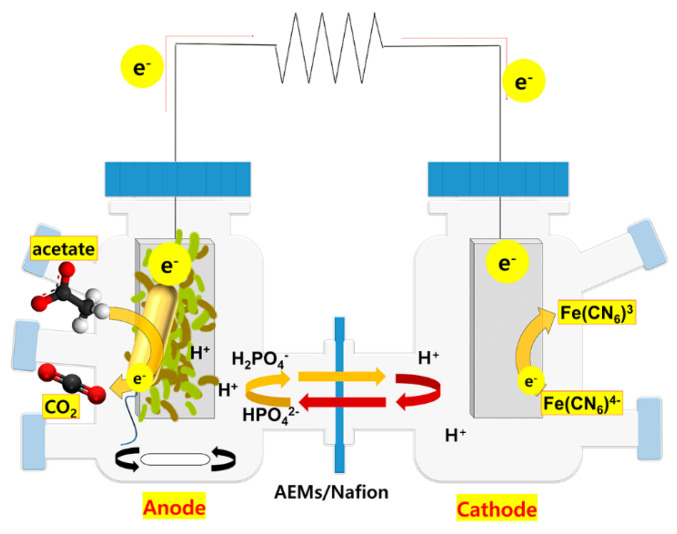
Schematic diagram of a dual-chambered MFC.

The operating conditions of MFC are different from the conventional fuel cell in terms of low temperature and use of bacterial media containing diverse ionic species. The H-type dual-chamber MFC reactors were used for electricity generation through the electroactive microbe with the configuration of the reactor assembled as shown in Figure 1. Briefly, the 250 mL of the glass bottle was used for the anode and cathode chamber with a glass side-arm (13 mm diameter) for reference electrode (Ag/AgCl, 3 M KCl) and a connection glass tube (21 mm diameter). The carbon felt (40 mm × 50 mm; NARA Cell-tech Co., Korea) electrodes were used as the anode and the cathode. A total of five MFC reactors were prepared by clamping different membranes in between the glass bottles. 

The anode chamber in the MFC was inoculated with the anaerobic secondary digester sludge (10% *v*/*v*), sampled from a wastewater treatment plant (Suyeong WWT Plant, Busan, Korea). The media in both chambers consisted of a mixture of 50 mM phosphate buffer (pH 7.0) with CH_3_COONa (3.28 g/L), NH_4_Cl (0.23 g/L), NaCl (0.04 g/L), MgSO_4_·H_2_O (0.01 g/L), and KCl (0.02 g/L). Additionally, the anolyte was added with the yeast extract (0.02 g/L) for the supplement of the unknown mineral while the catholyte was added potassium ferricyanide (0.1 M, 0.323 g/L) for the reduction reaction of protons. Finally, the reactors were cultured at 30 °C in a thermostatic room. 

The MFCs were cultured with the designed potassium phosphate buffer with anaerobic digest sludge till electricity generation. After pre-cultured, media replaced for operation. The voltage as electricity generated monitored minute-wise by a computer data logging system (LabVIEW™, National Instruments™, Austin, TX, USA) using a data acquisition board (NI DAQ USB-6218, National Instruments™, Austin, TX, USA). For comparison of the membrane performance, each MFCs connected with 500 Ω external resistance were compared at maximum voltage generation of stabilization range after over two cycles in the thermostatic environment. After stabilizing the MFCs, the obtained voltages were from 0.40 to 0.45 V in CCV (close circuit voltage), and 0.71 to 0.77 V on the OCV (open circuit voltage). These obtained voltages were similar, or superior in comparison to the previous reports [22]. To measure the maximum power density, the polarization data were measured by linear sweep voltammetry (LSV) tool using a potentiostat (VersaSTAT3, AMETEK, Oak Ridge, TN, USA). The Versa Studio™ software was used to scan the whole range starting from the maximum open-circuit voltage (OCV) at the rate of 10 mV/s

## 3. Results and Discussions

In comparison to random copolymers, multiblock copolymers are advantageous in terms of improved ion transport property with lower water content. The multiblock copolymers can self-assemble into well-defined hydrophobic/hydrophilic phase separation where the hydrophilic domain creates pathways for ion transport while the hydrophobic domains provide mechanical stability. Thus, membrane properties like conductivity, water uptake, and mechanical strength can be accurately controlled by changing the block length and their chemistry. However, the synthesis of the multiblock copolymer is more difficult than random copolymer because it involves a careful synthesis of oligomers with end-groups for a successful coupling reaction. To overcome this problem, we recently synthesized a multiblock copolymer with a convenient method of using highly reactive HFB as a linkage group [12]. Due to the high reactivity, HFB facilitates low-temperature coupling reaction which prevents trans-etherification leading to randomization of blocks. Therefore, multiblock copolymers with different cationic groups were synthesized using HFB in this study as shown in Scheme 1. The synthesis involves two steps: (1) synthesis of precursor multiblock copolymer, and (2) modification of the precursor multiblock copolymer with bromomethylation and subsequent quaternization. The detailed synthesis and characterizations have been discussed in the following sections.

### 3.1. Synthesis and Characterizations of the Oligomers and the Precursor Multiblock Copolymer 

The precursor multiblock copolymer, cardo-PES was synthesized from the coupling of tBPA-based precursor hydrophilic block, PES-1, and the PA cardo-group- based hydrophobic block, PES-3 as shown in Scheme 1. The tBPA contains benzylmethyl groups which provide bromomethylation with NBS and the Menshutkin reaction of bromomethylene groups can yield aggregated cation groups in the multiblock copolymer structure. The PA in the multiblock provides improved thermomechanical property which is essential under wet application conditions. Thus, the PES-1 and PES-2 were first synthesized in a K_2_CO_3_ mediated aromatic nucleophilic substitution reaction in NMP by controlling the monomer feed ratio. A certain excess molar amount of tBPA and PA were used for PES-1 and PES-2 oligomers, respectively for the -OH end-groups. The reaction was carried out by first heating at ~150 °C for 4 h for the removal of water as an azeotrope mixture with toluene and then, at 180 °C for 20 h. The powder polymer products were isolated after precipitating in excess methanol, washing, and drying under vacuum. The PES-1 and PES-2 oligomers were characterized by ^1^H NMR spectroscopy and GPC. The average oligomer length was calculated by comparing the proton integrals between the terminal phenyl groups (H7 for PES-1, H32 for PES-2) and the repeating unit (H5 for PES-1, H21 for PES-2) in the ^1^H NMR spectra (Figures S1 and S2a). The results of the ^1^H NMR spectrum analysis and the GPC are listed in Table 1. The good matching between the number average molecular weight of GPC and NMR spectrum confirmed the successful synthesis of -OH terminated oligomers.

The PES-3 was synthesized with –F termini by reacting the PES-2 with HFB in a K_2_CO_3_-mediated aromatic nucleophilic reaction in DMAc. Due to the volatile and high reactivity of HFB, an excess amount of HFB (1:10) was used for the reaction at a low temperature to avoid intermolecular oligomer coupling the reaction. The powder polymer products were isolated after precipitating in excess methanol, washing, and drying under vacuum. The complete disappearance of the terminal phenoxide proton peaks at 6.68 ppm in the ^1^H NMR spectrum (Figure S2b) confirmed the HFB end-capping of PES-2. In the ^19^F NMR, fluorine peaks with the integral ratios in agreement with their chemical structure also confirmed the HFB end-capping. 

**Figure 2:**
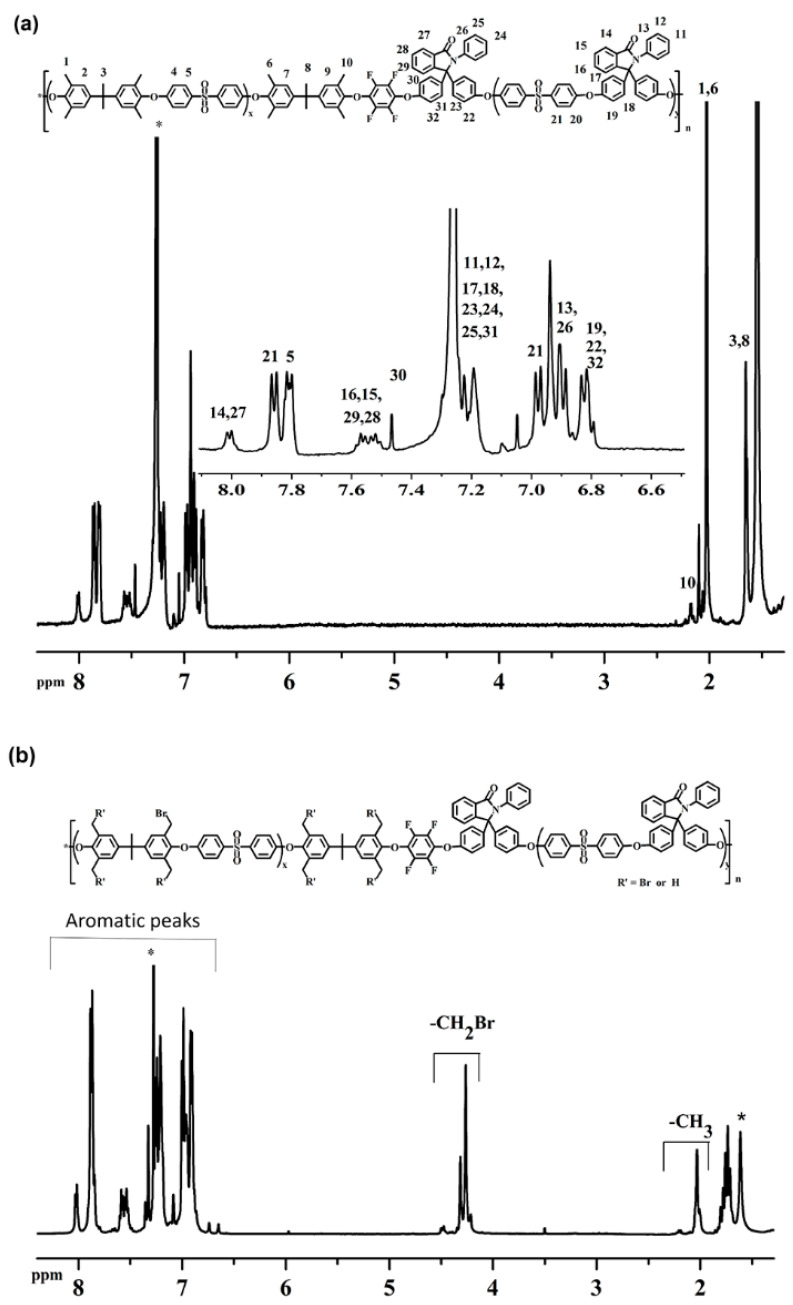
^1^H NMR spectra of (**a**) cardo-PES and (**b**) cardo-PES-Br in CDCl_3_ at 25 °C. * indicates solvent and moisture peaks.

The cardo-PES was synthesized in a base mediated 1:1 polycondensation reaction between the –OH (PES-1) and -F (PES-3) terminated oligomers (Scheme 1) in DMAc. Cyclohexane was used for azeotropic removal of the water in the reaction. The reaction just at 110 °C led to a viscous solution due to the high reactivity of terminal HFB. The low-temperature reaction prevents the possible randomization of the oligomer blocks through trans-etherification in high-temperature reactions [20]. The cardo-PES was isolated as a fibrous polymer after precipitation in acidified methanol, washing and drying under vacuum. In GPC (Figure S3), the synthesized cardo-PES showed a unimodal peak at the high average molecular weight region (Mw~118 kDa, Mn~27 kDa, PDI~4.3) in comparison to the reacting oligomers indicating the formation of the multiblock structure. The chemical structure of the cardo-PES was characterized by ^1^H NMR spectroscopy. In Figure 2a, the peaks were well-assigned corresponding to the starting oligomers. The complete disappearance of the PES-1 end group moieties in the alkyl region (2.2 and 2.0 ppm) indicated full coupling through HFB to form the multiblock copolymers. The ^19^F NMR spectrum revealed para coupling as a major reaction along with certain ortho or meta couplings. We assume that a small amount of ortho or meta reaction would have a little effect on the membrane property. Nevertheless, the copolymer composition matched well with the oligomer feed ratio supporting the multiblock structure of the cardo-PES. The synthesized cardo-PES showed good solubility in aprotic polar solvents (N-methyl pyrrolidone (NMP), dimethyl sulfoxide (DMSO), dimethylformamide (DMF), dimethylacetamide (DMAc), and tetrahydrofuran (THF)) and halogenated solvents (chloroform, dichloromethane, 1,1,2,2–tetrachloroethane (TCE)).

### 3.2. Synthesis and Characterization of the Brominated and Quaternized Polymers

Bromination of the cardo-PES was carried out using NBS (1.0 equiv. to ArCH_3_) as the brominating agent in presence of a radical initiator, AIBN in TCE at 85 °C. Unlike the crosslinking side reactions in chloromethylation, NBS/AIBN provides regioselective bromination of the benzylmethyl groups. Bromination reaction of cardo-PES using NBS/AIBN for 5 h resulted in a red color homogenous solution. The brominated polymer, cardo-PES-Br was isolated as a white-yellowish fibrous product after precipitation in ethanol, washing and drying. In the ^1^H NMR spectrum (Figure 2b), the cardo-PES-Br showed aromatic peaks corresponding to the cardo-PES. However, a new signal appeared at 4.29 ppm corresponding to the bromomethylene protons with the simultaneous weakening of the benzylmethyl proton signal at 2.1 ppm indicating selective benzyl bromination. The degree of bromination was calculated as 72.9% from the relative integral ratios of the bromomethylene and benzylmethyl peaks (Table 1). The bromination yield was also calculated as 72.9% (from the ratio of the amount of bromine detected in cardo-PES-Br and feed molar amount of NBS). As boromomethylene group can undergo an easy Menshutkin reaction, the controlled bromination by NBS/AIBN provides a more convenient route to tune the IECw in AEMs.

**Figure 3:**
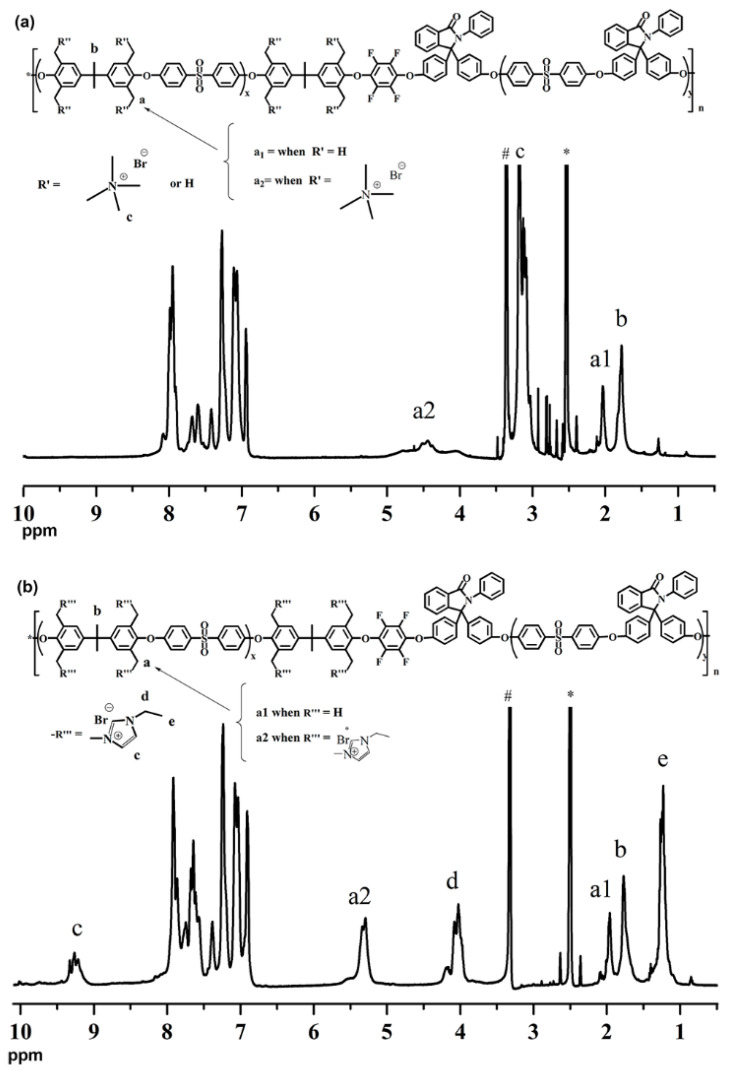
^1^H NMR spectra of (**a**) cardo-PES-QA and (**b**) cardo-PES-IM in DMSO-d_6_ at 25 °C. * indicates solvent peak and # indicates moisture peak.

The cardo-PES-QA and cardo-PES-IM were synthesized by the Menshutkin reaction of the bromomethylene groups in the cardo-PES-Br with TMA and 1-ethyl imidazole, respectively. The cardo-PES-QA and cardo-PES-IM were characterized by ^1^H NMR spectroscopy. In the ^1^H NMR spectrum of cardo-PES-QA (Figure 3a), the comparison of the signal at 3.2 ppm assigned to the -QA methyl with the aromatic peaks indicated successful quaternization with TMA. The sharp bromomethylene signal at 4.29 ppm in cardo-PES-Br became a broad signal at 4.5 ppm in cardo-PES-QA due to the strong electron-withdrawing effect. In the ^1^H NMR spectrum of cardo-PES-IM (Figure 3b), the shift of bromomethylene peak at 4.29 to ~5.4 ppm suggested successful complete imidazolium functionalization. The peaks at 9.3 and 4.1 ppm corresponding to the imidazolium protons (Hc) and benzyl protons (Hd) of the ethyl imidazole moiety confirmed the incorporation of imidazolium groups in the multiblock copolymer.

**Figure 4:**
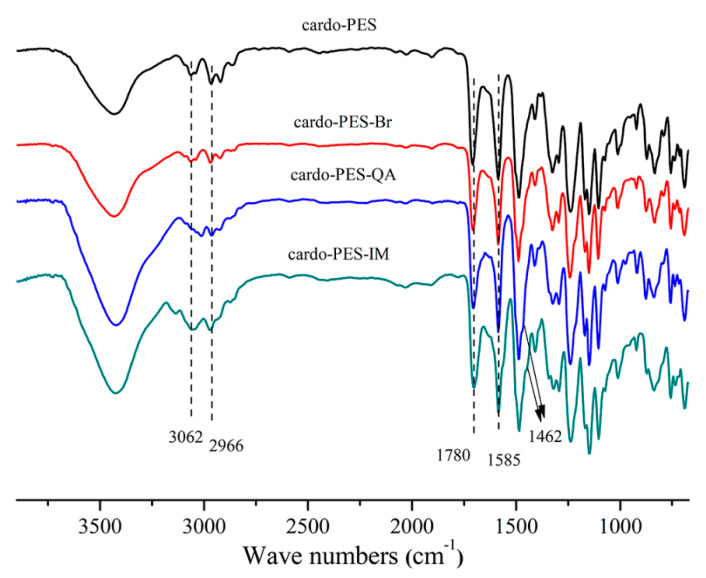
FT-IR spectra of the multiblock copolymers.

The cardo-PES-QA and cardo-PES-IM copolymers were also analyzed by FT-IR spectroscopy. The FT-IR spectra in Figure 4 show the aryl ether stretching peaks at 1245 cm^−1^ that are formed during the precursor polymerization. The sulfone (SO_2_) peaks appeared at 1317 and 1151 cm^−1^. The sharp peak at 1707 cm^−1^ was due to the imide type carbonyl stretching in the PA moieties. The stretching frequencies at 3066 and 1589 cm^−1^ correspond to the aromatic C–H and C=C stretching, respectively. The peaks at 2880–2995 cm^−1^ were due to the -CH_2_- and -CH_3_ stretching. The vibrational peaks at around 1562 and 756 cm^−1^ were ascribed to the imidazolium groups. The weak peak at 1462 cm^−1^ was attributed to the vibration of the C−N bond on the quaternary ammonium group or imidazolium group [23]. A wideband appeared at around 3400 cm^−1^ due to the stretching vibration of the O–H bond (from the absorbed moisture).

The cardo-PES-QA and cardo-PES-IM were soluble in various organic solvents such as DMF, DMAc, DMSO, and NMP [tested as 10% (*w*/*v*)]. Thus, membrane fabrication was possible by the solvent casting method. Under the homogeneous solution conditions, the thermodynamic driving forces can lead the self-assembly to phase-separated morphology which influences the membrane properties 

### 3.3. Thermal Stability

**Figure 5:**
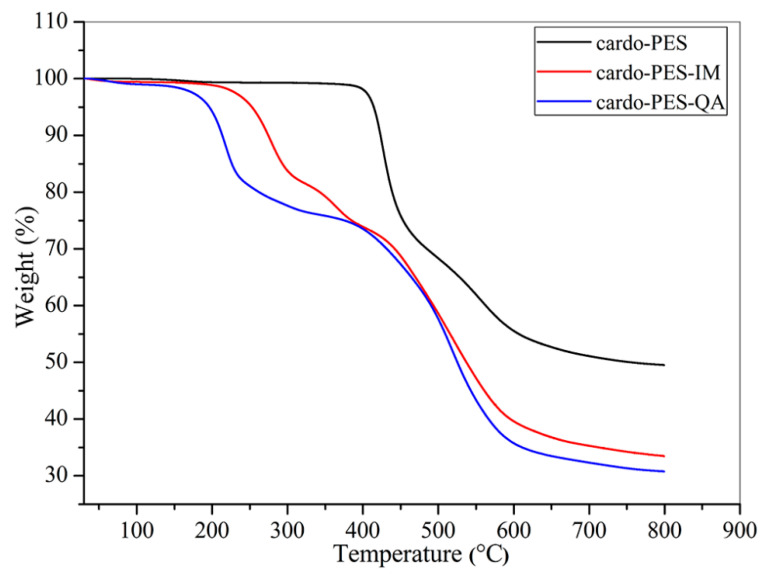
TGA thermograms of the multiblock copolymers.

The thermal stability of the cardo-PES, cardo-PES-QA and cardo-PES-IM (in Br^−^ form) was tested by TGA in the N_2_ atmosphere. The TGA thermogram is shown in Figure 5. The precursor copolymer, cardo-PES showed single step degradation corresponding to the back-bone decomposition with the 5 wt% loss temperature, T_d5%_ of ca. 412 °C. On the other hand, the ionomeric copolymers exhibited distinct two-step degradation profiles. The first weight loss was due to the loss of the ionic groups (onset decomposition temperature, T_d-onset_: ~194 °C, cardo-PES-QA, and ~ 242 °C, cardo-PES-IM). The second weight-loss matching with the cardo-PES indicated the back-bone decomposition. The T_d5%_ was ca. 200 °C for cardo-PES-QA and ca. 250 °C for cardo-PES-IM. The higher thermal stability of cardo-PES-IM than cardo-PES-QA was due to the aromatic structure of the imidazolium moieties.

### 3.4. Morphology

**Figure 6:**
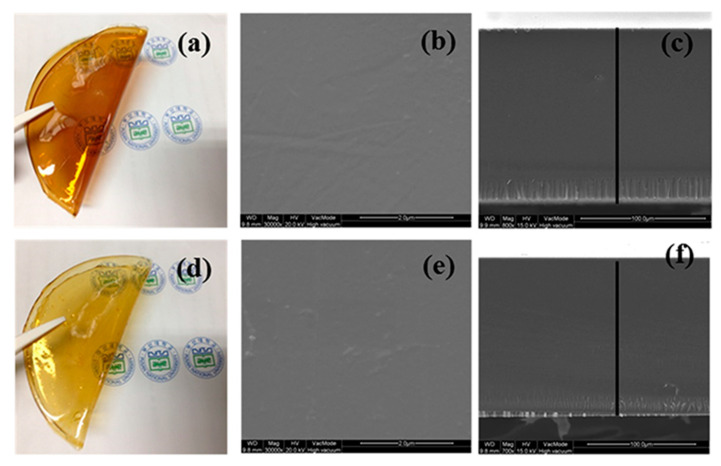
Photographs, surface, and cross-sectional SEM images of the cardo-PES-QA (**a**–**c**) and cardo-PES-IM (**d**–**f**) membranes, respectively.

The cardo-PES-QA and cardo-PES-IM yielded transparent and flexible membranes (Figure 6a,d). In the SEM, the cardo-PES-QA and cardo-PES-IM membrane surface appeared smooth and uniform (Figure 6b,e). However, the cross-sectional image of cardo-PES-IM (Figure 6c) appeared tighter and smoother than cardo-PES-QA (Figure 6f). This distinct feature might be due to the more hydrophilic nature of the QA groups [12,23]. 

**Figure 7:**
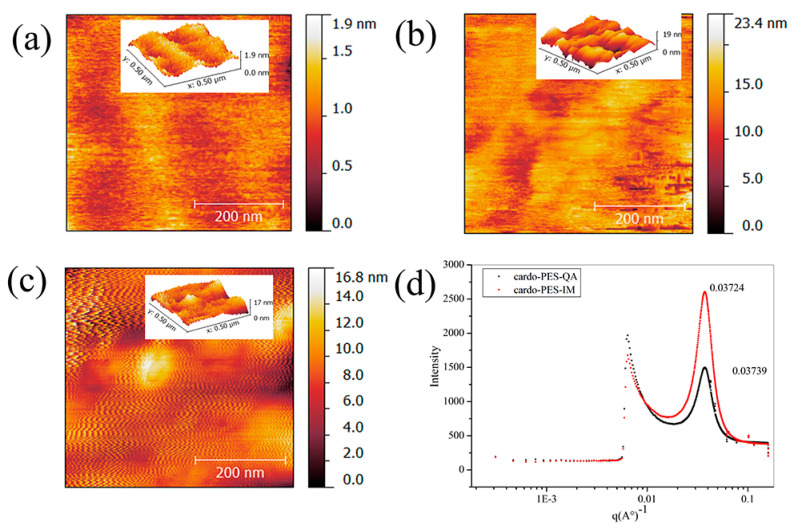
AFM phase images of the cardo-PES-QA (**a**) and cardo-PES-IM (**b**,**c**) membranes. (**b**) Membrane equilibrated at 50% RH, and (**c**) fully wet membrane dried at 50 °C for 30 min. Inset pictures indicate the corresponding 3D images. (**d**) SAXS profiles of the multiblock copolymer AEMs.

The morphology of the copolymer membranes in particularly the amphiphilic copolymers depends on various parameters like composition, molecular weight, solvent type, solution concentration, rate of solvent evaporation, shear stress, and film thickness [24,25]. Hence, the ionomeric membranes were scanned by AFM to investigate the hydrophilic/hydrophobic phase-separated morphology. Figure 7 shows the AFM topographic images of the membrane surfaces recorded on the size scale of 500 × 500 nm^2^ in a tapping mode. The darker regions represent the soft hydrophilic domains while the bright regions represent the hard hydrophobic domains. The block structure of the copolymers induced distinct phase-separated morphology in the membranes. The AFM images of both the ionomer membranes showed hydrophilic and hydrophobic domains of several hundreds of nanometers distributed nonhomogeneously on the membrane surface. The cardo-PES-QA in Figure 7a showed relatively phase-separated finely corrugated structures than the cardo-PES-IM in Figure 7b. The corrugation of the cardo-PES-IM membrane surface was slightly larger than that of cardo-PES-QA. Figure 7b,c shows the height images of cardo-PES-IM at two different conditions: (i) the membrane equilibrated at 50% RH, and (ii) fully wet membrane dried at 50 °C for 30 min. When compared to Figure 7a,b, Figure 7c revealed a more uniformly orderly corrugated phase-separated lamellar structure of the cardo-PES-IM indicating reformation of the hydrophobic/hydrophilic phase morphology with higher water absorption. 

The cardo-PES-QA and cardo-PES-IM (Br- from) membranes pre-equilibrated at 75% RH were also analyzed for morphologies by SAXS. The results are presented by the intensity versus the scattering vector, *q* plot as shown in Figure 7. In Figure 7d, q represents the characteristic separation length between the ionic domains distributed in the hydrophobic domains of the membrane. The AEMs showed distinct ionomeric peaks at q_max_~0.37 nm^−1^, corresponding to d~16.9 nm. This indicates similar ion cluster formation due to the precursor block structure of the ionomers. However, the relatively narrower peak profile for cardo-PES-IM indicates the uniform distribution of ionic domains, while the broader peak of cardo-PES-QA demonstrates a wider distribution of periodicities. Overall, well-defined hydrophilic/hydrophobic phase-separation with inter-connected ionic hydrophilic domain in the cardo-PES-QA and cardo-PES-IM AEMs was assumed to facilitate the ion conductivity while maintaining dimensional stability.

### 3.5. Membrane Properties

The theoretical IECw value of the AEMs was calculated using the block copolymer composition and degree of bromination with the assumption of 100% quaternization of methylene bromide groups. The theoretical IECw values of the cardo-PES-QA and cardo-PES-IM membranes were 1.54 and 1.42 mequiv./g, respectively. The difference in the IECw values is due to the difference in the masses of QA and IM groups. The IECw of the AEMs was also measured using Mohr’s method by the titration of Br^-^ with silver nitrate. The values are summarized in Table 2. The experimental values were above 95% of the theoretical values. The minute difference between the values might be due to the calculation of polymer mass.

**Figure 8:**
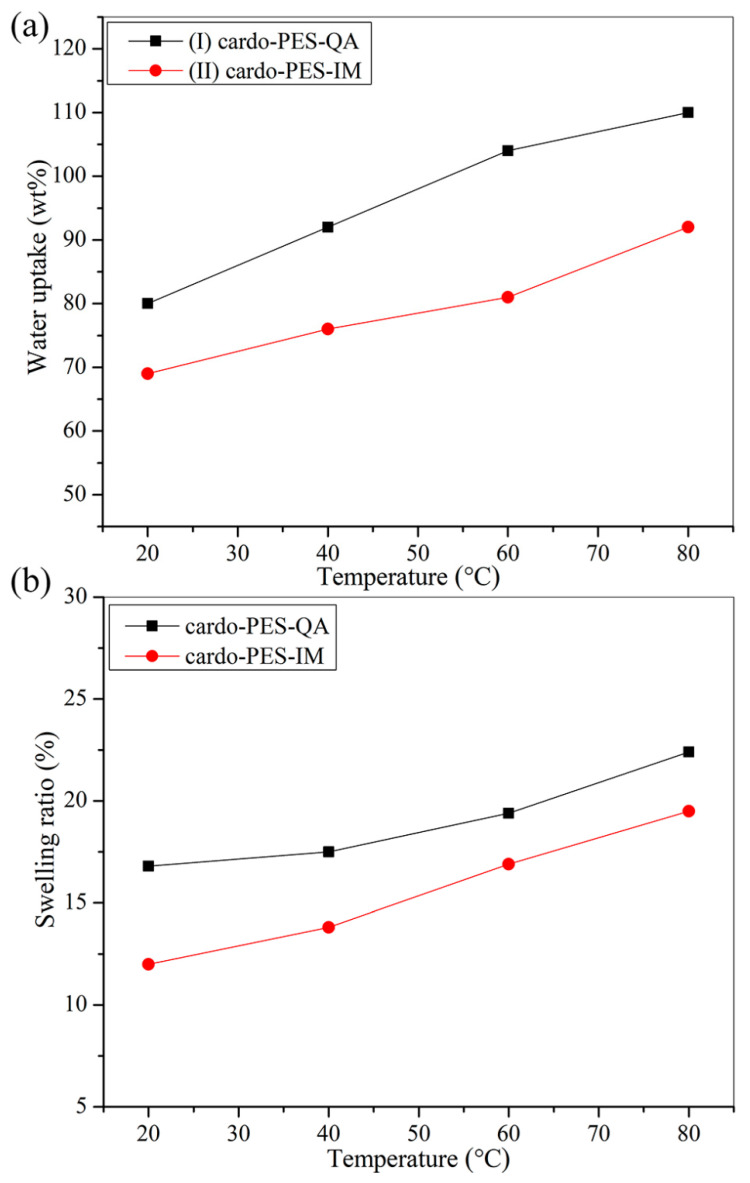
(**a**) Water uptake and (**b**) swelling ratio of the AEMs as a function of temperature.

Table 3 summarizes the water uptake, swelling ratio (longitudinal, Δl and thickness, Δt) and hydroxide conductivity of the AEMs. Figure 8a shows the temperature dependence of water uptake of the AEMs. The water uptake increased with the increase in temperature and IECw as per the expectation. The difference in the water uptake between the membranes became larger as the temperature increased. The membranes also followed a similar swelling trend as the water uptake (Figure 8b). The swelling of the AEMs was well below 25% over the temperature range. Thus, the synthesized AEMs showed reasonably similar or higher water uptake and swelling in comparison to the previously reported membranes [14,23,26,27,28,29,30].

**Figure 9:**
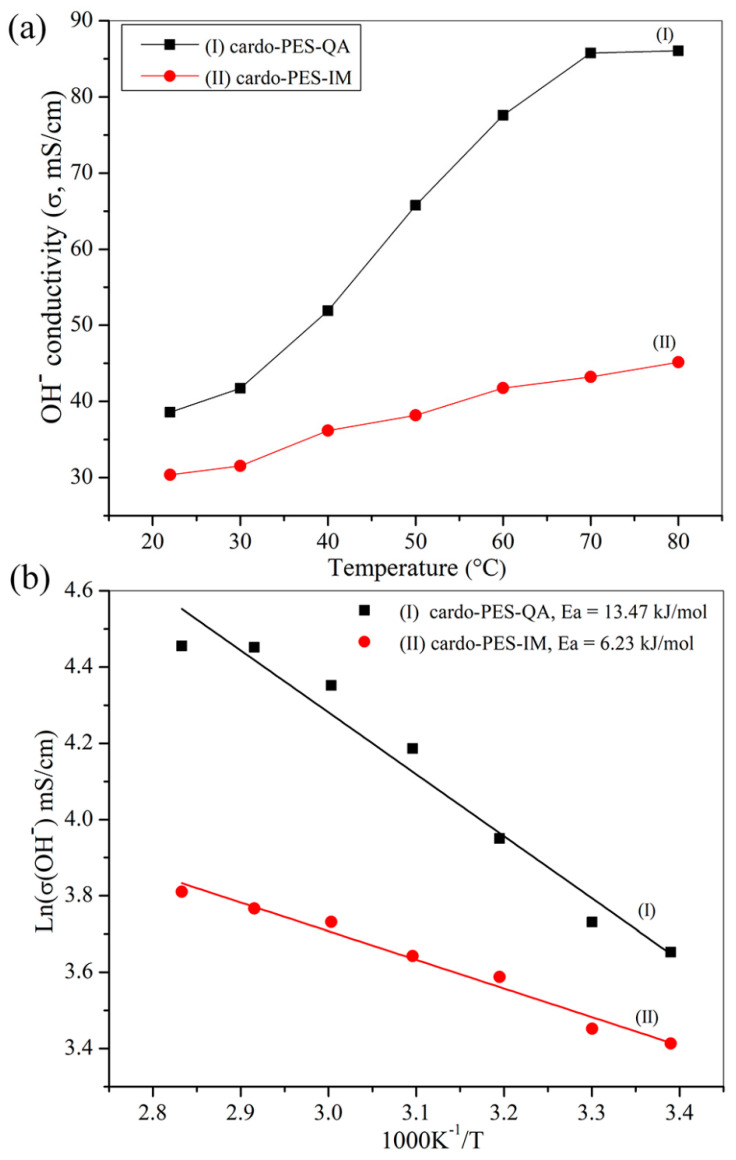
(**a**) The OH^−^ conductivity as a function of temperature and (**b**) Arrhenius temperature dependence of OH^−^ conductivity (σ) of the AEMs.

A lot of research has shown that the hydrophilic/hydrophobic phase-separated morphology in ionomeric membranes, particularly PEMs, influences the membrane properties including ion conductivity with efficient utilization of the absorbed water [14,31,32]. It is also known that AEMs if not driven by polymer structure do not generally show well-defined phase-separated morphology, unlike PEMs. Thus, we previously reported that the well-defined phase-separated membrane morphology induced by the block copolymer structure of AEMs resulted in higher ion conductivity with controlled water uptake [12]. Thus, we measured the ion conductivity to know the transport property of the synthesized block copolymer AEMs. The ion conductivity of AEMs was measured in three different counter ion forms (OH^−1^, HCO_3_^−1^, and Cl^−1^). For this, the membranes were first converted to OH^−1^_,_ HCO_3_^−1^, and Cl^−1^ forms by ion exchange in 1 M NaOH, 1 M NaHCO_3_, and 1 M NaCl solutions, respectively for 24 h. The in-plane ion conductivity (σ) of the AEMs was measured in deionized water as a function of temperature (30–80 °C) by impedance spectroscopy. As expected, the ionic conductivity of the synthesized AEMs increased with increasing IECw and temperature (Figure 9a and Figure S4). 

At higher temperature, the higher local mobility of water with certain perturbation in chain structures creates long-range ion rich percolated structures that facilitated ion transportation in the membrane resulting in higher ion conductivity. The AEMs exhibited hydroxide conductivities of 30–38 mS/cm at 20 °C (and 45–86 mS/cm at 80 °C). The lower ion conductivity of cardo-PES-IM than cardo-PES-QA is due to the lower IEC, lower water uptake, and lower basicity of imidazolium groups [33]. The conductivity values were comparable or higher than the previously reported AEMs at similar IEC [14,23,26,27,28,29,30,31,34,35,36,37,38,39,40,41,42,43,44,45]. Table 4 lists the IEC and hydroxide conductivity of previously reported different AEMs. This proves that the block structure of the AEMs with a well-defined phase-separated structure (as confirmed by SAXS) contributed to higher hydroxide conductivity. As air exposure can decrease the OH^−1^ conductivity of the membrane due to the rapid neutralization of OH^−1^ with atmospheric CO_2_ to bicarbonate of lower dilute solutions mobility [23,46,47], the conductivity of the AEMs was further measured in chloride and bicarbonate forms. The AEMs showed HCO_3_^−1^ conductivity of 7.8–9.5 mS/cm at 20 °C (and 30.9–42.2 mS/cm at 80 °C), and Cl^−1^ conductivity of 7.1–12.6 mS/cm at 20 °C (and 37.2–60.1 mS/cm at 80 °C). The Cl^−1^ and HCO_3_^−1^ conductivity was approximately one third and one fourth of the OH^−1^ conductivity. The difference was reasonable when considered the individual ion mobilities (ν (OH^−1^) = 2.0 × 10^−7^, ν (Cl^−1^) = 7.9 × 10^−8^, and ν (HCO_3_^−1^) = 4.6 × 10^−8^ m^2^s^−1^V^−1^ in infinitely diluted aqueous solution at 298 K) [17]. Figure 9b and Figure S5 show the approximate Arrhenius-type temperature-dependence of ion conductivity of the AEMs. The apparent activation energy (Ea) for the membrane hydroxide conductivity was calculated by a linear least-square fit of the data presented in Figure 9b according to the Arrhenius equation: $\sigma =Ae^{\frac{E_{a}}{RT}}$ (σ is the hydroxide conductivity (mS/cm), R is the universal gas constant (8.314 J/mol K), A is the pre-exponential factor, and T is the absolute temperature (K)). The apparent Ea for OH^−1^ conductivity was 13.47 kJ/mol for cardo-PES-QA and 6.23 kJ/mol for cardo-PES-IM (cardo-PES-QA: 21.78 kJ/mol, Ea of HCO_3_^−1^ conductivity, 24.19 kJ/mol, Ea of Cl^−1^ conductivity; cardo-PES-IM: 20.37 kJ/mol, Ea of HCO_3_^−1^ conductivity, 25.27 kJ/mol, Ea of Cl^−1^ conductivity). The reason for higher Ea values of HCO_3_^−1^ (or Cl^−1^) conductivity than OH^−1^ (or H^+^) conductivity is that HCO_3_^−1^ does not undergo hopping transport which results in lower dilute solution ionic mobility [47]. The higher Ea value of cardo-PES-QA indicated that its ion mobility is strongly dependent on temperature in comparison to the cardo-PES-IM. The lower Ea value of the cardo-PES-IM may be due to the somewhat better phase-separated morphology with connected hydrophilic domains which provides less barrier to the ion movements even at lower water absorption. Overall, the Ea values were lower or similar to the previously reported AEMs (9–25 kJ/mol) [28,30,38,39,47,48,49], suggesting the synthesized AEMs share a similar conduction mechanism involving hydrated ions.

### 3.6. Microbial Fuel Cell Performance

**Figure 10:**
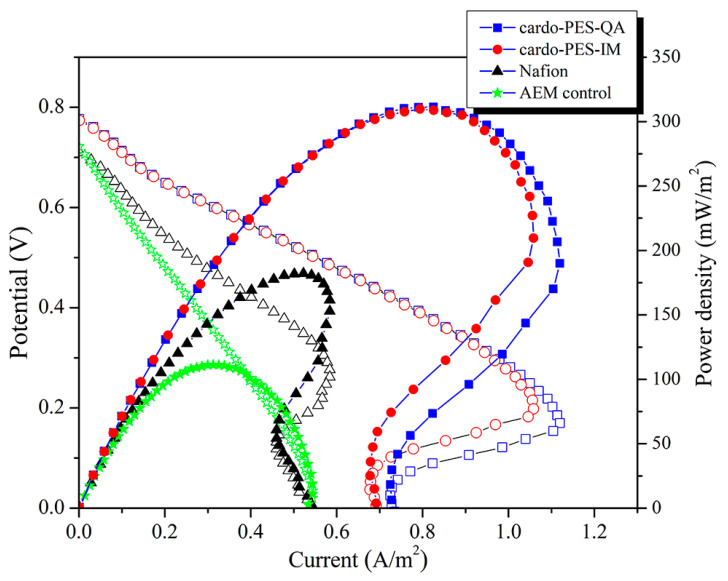
Microbial fuel cell performance of the AEMs and the commercial membranes (solid symbols represent the power density while the corresponding blank symbols represent the potential).

The synthesized cardo-PES-QA and cardo-PES-IM membranes measured the MFC performances in terms of the power density due to the electricity generation and compared with the CEM, Nafion, and the AEM, FAB-PK-130. The MFC results are shown in Figure 10. The MFC with the synthesized AEMs obtained slightly higher voltage generation as OCV up to 0.77 V than Nafion (0.71 V) and FAB-PK-130 (0.72 V). However, the synthesized AEMs showed significantly higher power density than Nafion and FAB-PK-130. The cardo-PES-QA and cardo-PES-IM achieved a similar power density as 311.39 and 309.69 mW/m^2^ (at 0.82 A/m^2^), respectively. Thus, the synthesized membranes drive approximately 2.8 times higher power density than FAB-PK-130 with a power density of 111.13 mW/m^2^. Meanwhile, the Nafion obtained a higher power density of 182.15 mW/m^2^ which was 1.6 times higher than FAB-PK-130 and ~1.7 times lower than the synthesized AEMs. These results indicate that the MFC achieved higher proton and electron movement with the synthesized AEMs than Nafion and FAB-PK-130. Like Nafion, the synthesized AEMs showed power overshoots at higher current densities of the power density curve due to insufficient biofilm acclimation and high mass transport limitation on the electrode surface. Zhu et al. [50] reported that the power overshoot of MFCs incubated at the lowest anode potential was associated with decreasing electroactivity of the anodic biofilm at the high potential region, which resulted from the lack of sufficient electron transfer components to shuttle electrons at rates needed for these more positive potentials. Thus, these overshoot curves were because of so unstable performance of the MFC at high current densities.

## 4. Conclusions

In summary, a new series of phenolphthalein-based multiblock poly(arylene ether sulfone) AEMs were synthesized by block polycondensation, bromination, and quaternization. The bulky aromatic structures of phenolphthalein moieties on the hydrophobic segments and the hydrophobic HFB linkage induced a flexible tough membrane with well-defined phase-separated morphology for ion transport. The cardo-PES-IM AEM having combined benefits of both the phase-separated morphology of the block copolymer and the thermochemical stability of the imidazolium functionality offers good thermal stability, dimensional and chemical stability. The cardo-PES-IM showed reasonably higher conductivity, was however lower in comparison to cardo-PES-QA due to low IEC value (cardo-PES-IM, IEC = 1.38 mequiv./g; cardo-PES-QA, IEC = 1.49 mequiv./g). The OH^−1^ ionic conductivity of the membranes was in the range of 30–45 mS/cm for cardo-PES-QA and 38–86 mS/cm for cardo-PES-IM. Finally, the synthesized AEMs were checked as separator in dual-chambered microbial fuel cell and compared their performance with Nafion and FAB-PK-130. The AEMs showed quite higher power output in comparison to the commercial membranes. However, to maximize the power output, other variables in the fuel cell need to be further optimized.

## Figures and Tables

**Table 1:** Composition and physical properties of the polymers.

Polymers	M_n_^target^ (Da) /DP	M_n_^NMR^ (Da) /DP	M_n_^GPC^ (Da) /DP	PDI ^a^
PES-1	4000/8	5140/10	5110/10	1.67
PES-2	8000/13	8680/14	8060/13	1.55
PES-3	-	-	8480	1.65
Cardo-PES	-	-	27,200	4.34

^a^ PDI, polydispersity index.

**Table 2:** Bromination and IEC of the polymers.

Bromination Yield ^a^ (%)	DBM ^b^ (%)	IEC_W_ (mequiv./g)			
		Cardo-PES-QA		Cardo-PES-IM	
		Theo. ^c^	Titr. ^d^	Theo. ^c^	Titr. ^d^
72.9	72.9	1.54	1.49	1.42	1.38

^a^ Calculated from the bromine added in NBS and bromine detected in the cardo-PES-Br in ^1^H NMR spectrum. ^b^ degree of bromination calculated from ^1^H NMR spectrum by comparing methylene (-CH_2_Br) to methyl (-CH_3_) protons. ^c^ Calculated from the block copolymer composition and degree of bromination determined from ^1^H NMR spectrum. ^d^ Calculated using Mohr’s method of titration.

**Table 3:** Anion exchange membrane properties of cardo-PES-QA and cardo-PES-IM copolymers.

Polymer	IEC_W,theo_ (mequiv./g)	WU_W_ (%)		λ	Δl (%)	Δt (%)	σ (mS/cm)		E_a_^a^ (KJ/mol)
		20 °C	80 °C	20 °C	20 °C	20 °C	20 °C	80 °C	
Cardo-PES-QA	1.54	80	110	28.8	16.8	25.2	38	86	13.46
Cardo-PES-IM	1.42	69	92	26.9	12	16.7	30	45	6.23

^a^ Activation energy determined in the temperature range: 30–80 °C and heating rate 1–2 °C/min.

**Table 4:** IEC and hydroxide conductivity of different AEMs.

Membrane	Ionic Group	IEC (mequiv./g)	Conductivity (mS/cm)
Cardo-PES-QA [This work]	QA	38	38 at 20 °C
Cardo-PES-IM [This work]	IM	30	30 at 20 °C
QPE-X16Y11 [14]	QA	1.38	52 at 60 °C
PSf80-ImOH [26]	IM	1.39	16.1 at 20 °C
QRPES-40 [35]	QA	1.50	21 at room temperature
IM-PFEKS [23]	IM	1.64	17.1 at room temperature
ATMPP [27]	QA	1.57	50 at 30 °C
PSF-PTMA(X62) [36]	QA	146	33 at 20 °C
QPAE-d [38]	QA	1.43	5.8 at 20 °C
PSf-ImOH 90% [28]	IM	1.58	~20 at 20 °C
QPAE-X25Y21 [39]	QA	1.45	16.9 at 20 °C
NAPEK-Q-100 [40]	QA	1.46	23 at 20 °C
mPES-X_9_._2_Y_3_._4_ [41]	QA	1.45	4.9 at 25 °C
EI-PES [42]	IM	1.45	30 at 20 °C
bQAPDHTPE-OH20 [29]	QA	1.66	21.37 at 30 °C
AMPE-M15N15 [43]	QA	1.33	34.4 at 30 °C
QPAES-X16Y10 [30]	QA	1.45	15.4 at 20 °C
PAES-Q-75 [44]	QA	1.49	21.9 at 25 °C
QPE-bl-5 (X9Y8) [45]	QA	1.5	~41 at 60 °C
ImPESN-9–22 [31]	IM	1.58	38.5 at 30 °C

## Data Availability

Not applicable.

## Supplementary Materials

The following are available online at https://www.mdpi.com/2077-0375/11/6/454/s1, Figure S1: ^1^H NMR spectrum of PES-1 recorded in CDCl_3_ at 25 °C, Figure S2: ^1^H NMR spectra of (a) PES-2 and (b) PES-3 recorded in CDCl_3_ at 25 °C, Figure S3: SEC traces of oligomers and precursor block copolymer, Figure S4: Temperature dependence of (a) Cl^−1^ and (b) HCO_3_^−1^ conductivity of the AEMs, Figure S5: Arrhenius temperature dependence of (a) Cl^−1^ and (b) HCO_3_^−1^ conductivity (σ) of the AEMs.

## References

[1] Sharma V., Kundu P. (2010). Biocatalysts in Microbial Fuel Cells. Enzym. Microb. Technol..

[2] Li W.-W., Sheng G.-P., Liu X.-W., Yu H.-Q. (2011). Recent Advances in the Separators for Microbial Fuel Cells. Bioresour. Technol..

[3] Xu W., Ledin P.A., Shevchenko V.V., Tsukruk V.V. (2015). Architecture, Assembly, and Emerging Applications of Branched Functional Polyelectrolytes and Poly(Ionic Liquid)s. ACS Appl. Mater. Interfaces.

[4] Pandit S., Khilari S., Bera K., Pradhan D., Das D. (2014). Application of PVA–PDDA Polymer Electrolyte Composite Anion Exchange Membrane Separator for Improved Bioelectricity Production in a Single Chambered Microbial Fuel Cell. Chem. Eng. J..

[5] Lin C.X., Wu H.Y., Li L., Wang X.Q., Zhang Q.G., Zhu A.M., Liu Q.L. (2018). Anion Conductive Triblock Copolymer Membranes with Flexible Multication Side Chain. ACS Appl. Mater. Interfaces.

[6] Rao A.H.N., Nam S.Y., Kim T.-H. (2015). Comb-Shaped Alkyl Imidazolium-Functionalized Poly(Arylene Ether Sulfone)s as High Performance Anion-Exchange Membranes. J. Mater. Chem. A.

[7] Gu S., Cai R., Yan Y. (2011). Self-Crosslinking for Dimensionally Stable and Solvent-Resistant Quaternary Phosphonium Based Hydroxide Exchange Membranes. Chem. Commun..

[8] Zhao C.H., Gong Y., Liu Q.L., Zhang Q.G., Zhu A.M. (2012). Self-Crosslinked Anion Exchange Membranes by Bromination of Benzylmethyl-Containing Poly(Sulfone)s for Direct Methanol Fuel Cells. Int. J. Hydrog. Energy.

[9] Morandi C.G., Peach R., Krieg H.M., Kerres J. (2014). Novel Morpholinium-Functionalized Anion-Exchange PBI–Polymer Blends. J. Mater. Chem. A.

[10] Niu M., Zhang C., He G., Zhang F., Wu X. (2019). Pendent Piperidinium-Functionalized Blend Anion Exchange Membrane for Fuel Cell Application. Int. J. Hydrog. Energy.

[11] Merle G., Wessling M., Nijmeijer K. (2011). Anion Exchange Membranes for Alkaline Fuel Cells: A Review. J. Membr. Sci..

[12] Mohanty A.K., Devaraju S., Kim N., Paik H.-J. (2018). Synthesis and Characterization of Poly(Ether Sulfone) Block Copolymers Containing Pendent Quaternary Ammonium- and Imidazolium Groups as Anion Exchange Membranes. Solid State Ion..

[13] Mohanty A.K., Sen S.K., Ghosh A., Maji S., Banerjee S. (2009). Synthesis, Characterization, and Comparison of Properties of New Fluorinated Poly(Arylene Ether)s Containing Phthalimidine Moiety in the Main Chain. Polym. Adv. Technol..

[14] Tanaka M., Fukasawa K., Nishino E., Yamaguchi S., Yamada K., Tanaka H., Bae B., Miyatake K., Watanabe M. (2011). Anion Conductive Block Poly(Arylene Ether)s: Synthesis, Properties, and Application in Alkaline Fuel Cells. J. Am. Chem. Soc..

[15] Li L., Wang Y. (2005). Quaternized Polyethersulfone Cardo Anion Exchange Membranes for Direct Methanol Alkaline Fuel Cells. J. Membr. Sci..

[16] Xiong Y., Liu Q.L., Zeng Q.H. (2009). Quaternized Cardo Polyetherketone Anion Exchange Membrane for Direct Methanol Alkaline Fuel Cells. J. Power Sources.

[17] Tanaka M., Koike M., Miyatake K., Watanabe M. (2010). Synthesis and Properties of Anion Conductive Ionomers Containing Fluorenyl Groups for Alkaline Fuel Cell Applications. Polym. Chem..

[18] Zhang Q., Zhang Q., Wang J., Zhang S., Li S. (2010). Synthesis and Alkaline Stability of Novel Cardo Poly(Aryl Ether Sulfone)s with Pendent Quaternary Ammonium Aliphatic Side Chains for Anion Exchange Membranes. Polymer.

[19] Rao A.H., Kim H.-J., Nam S., Kim T.-H. (2013). Cardo Poly(Arylene Ether Sulfone) Block Copolymers with Pendant Imidazolium Side Chains as Novel Anion Exchange Membranes for Direct Methanol Alkaline Fuel Cell. Polymer.

[20] Badami A.S., Lane O., Lee H.-S., Roy A., McGrath J.E. (2009). Fundamental Investigations of the Effect of the Linkage Group on the Behavior of Hydrophilic–Hydrophobic Poly(Arylene Ether Sulfone) Multiblock Copolymers for Proton Exchange Membrane Fuel Cells. J. Membr. Sci..

[21] Myung B.Y., Kim J.S., Kim J.-J., Yoon T.H. (2003). Synthesis and Characterization of Novel Polyimides with 2,2-bis[4(4-Aminophenoxy)Phenyl]Phthalein-3?,5?-Bis(Trifluoromethyl)Anilide. J. Polym. Sci. Part A Polym. Chem..

[22] Zhu L., Pan J., Wang Y., Han J., Zhuang L., Hickner M.A. (2016). Multication Side Chain Anion Exchange Membranes. Macromolecules.

[23] Chen D., Hickner M.A. (2012). Degradation of Imidazolium- and Quaternary Ammonium-Functionalized Poly(Fluorenyl Ether Ketone Sulfone) Anion Exchange Membranes. ACS Appl. Mater. Interfaces.

[24] Li Q., Chen Y., Rowlett J.R., McGrath J.E., Mack N.H., Kim Y.S. (2014). Controlled Disulfonated Poly(Arylene Ether Sulfone) Multiblock Copolymers for Direct Methanol Fuel Cells. ACS Appl. Mater. Interfaces.

[25] Mohanty A.K., Mistri E.A., Banerjee S., Komber H., Voit B. (2013). Highly Fluorinated Sulfonated Poly(Arylene Ether Sulfone) Copolymers: Synthesis and Evaluation of Proton Exchange Membrane Properties. Ind. Eng. Chem. Res..

[26] Zhang F., Zhang H., Qu C. (2011). Imidazolium Functionalized Polysulfone Anion Exchange Membrane for Fuel Cell Application. J. Mater. Chem..

[27] Hibbs M.R., Fujimoto C.H., Cornelius C.J. (2009). Synthesis and Characterization of Poly(Phenylene)-Based Anion Exchange Membranes for Alkaline Fuel Cells. Macromolecules.

[28] Yan X., He G., Gu S., Wu X., Du L., Wang Y. (2012). Imidazolium-Functionalized Polysulfone Hydroxide Exchange Membranes for Potential Applications in Alkaline Membrane Direct Alcohol Fuel Cells. Int. J. Hydrogen Energy.

[29] Hossain A., Lim Y., Lee S., Jang H., Choi S., Jeon Y., Lim J., Kim W.G. (2014). Comparison of Alkaline Fuel Cell Membranes of Random & Block Poly(Arylene Ether Sulfone) Copolymers Containing Tetra Quaternary Ammonium Hydroxides. Int. J. Hydrogen Energy.

[30] Li X., Liu Q., Yu Y., Meng Y. (2014). Synthesis and Properties of Multiblock Ionomers Containing Densely Functionalized Hydrophilic Blocks for Anion Exchange Membranes. J. Membr. Sci..

[31] Lai A.N., Wang L.S., Lin C.X., Zhuo Y.Z., Zhang Q.G., Zhu A.M., Liu Q.L. (2015). Phenolphthalein-Based Poly(Arylene Ether Sulfone Nitrile)s Multiblock Copolymers As Anion Exchange Membranes for Alkaline Fuel Cells. ACS Appl. Mater. Interfaces.

[32] Weiber E.A., Meis D., Jannasch P. (2015). Anion Conducting Multiblock Poly(Arylene Ether Sulfone)s Containing Hydrophilic Segments Densely Functionalized with Quaternary Ammonium Groups. Polym. Chem..

[33] Kwasny M.T., Zhu L., Hickner M.A., Tew G.N. (2018). Thermodynamics of Counterion Release Is Critical for Anion Exchange Membrane Conductivity. J. Am. Chem. Soc..

[34] Lin B., Qiu L., Qiu B., Peng Y., Yan F. (2011). A Soluble and Conductive Polyfluorene Ionomer with Pendant Imidazolium Groups for Alkaline Fuel Cell Applications. Macromolecules.

[35] Zhao Z., Wang J., Li S., Zhang S. (2011). Synthesis of Multi-Block Poly(Arylene Ether Sulfone) Copolymer Membrane with Pendant Quaternary Ammonium Groups for Alkaline Fuel Cell. J. Power Sources.

[36] Li N., Zhang Q., Wang C., Lee Y.M., Guiver M.D. (2012). Phenyltrimethylammonium Functionalized Polysulfone Anion Exchange Membranes. Macromolecules.

[37] Li X., Yu Y., Liu Q., Meng Y. (2012). Synthesis and Properties of Anion Conductive Ionomers Containing Tetraphenyl Methane Moieties. ACS Appl. Mater. Interfaces.

[38] Li X., Liu Q., Yu Y., Meng Y. (2013). Quaternized Poly(Arylene Ether) Ionomers Containing Triphenyl Methane Groups for Alkaline Anion Exchange Membranes. J. Mater. Chem. A.

[39] Li X., Yu Y., Liu Q., Meng Y. (2013). Synthesis and Properties of Anion Conductive Multiblock Copolymers Containing Tetraphenyl Methane Moieties for Fuel Cell Application. J. Membr. Sci..

[40] Liu Z., Li X., Shen K., Feng P., Zhang Y., Xu X., Hu W., Jiang Z., Guiver M.D. (2013). Naphthalene-Based Poly(Arylene Ether Ketone) Anion Exchange Membranes. J. Mater. Chem. A.

[41] Park D.-Y., Kohl P.A., Beckham H.W. (2013). Anion-Conductive Multiblock Aromatic Copolymer Membranes: Structure–Property Relationships. J. Phys. Chem. C.

[42] Rao A.H., Thankamony R.L., Kim H.-J., Nam S., Kim T.-H. (2013). Imidazolium-Functionalized Poly(Arylene Ether Sulfone) Block Copolymer as an Anion Exchange Membrane for Alkaline Fuel Cell. Polymer.

[43] Jasti A., Shahi V.K. (2014). A Facile Synthesis of Highly Stable Multiblock Poly(Arylene Ether)s Based Alkaline Membranes for Fuel Cells. J. Power Sources.

[44] Li X., Nie G., Tao J., Wu W., Wang L., Liao S. (2014). Assessing the Influence of Side-Chain and Main-Chain Aromatic Benzyltrimethyl Ammonium on Anion Exchange Membranes. ACS Appl. Mater. Interfaces.

[45] Yokota N., Ono H., Miyake J., Nishino E., Asazawa K., Watanabe M., Miyatake K. (2014). Anion Conductive Aromatic Block Copolymers Containing Diphenyl Ether or Sulfide Groups for Application to Alkaline Fuel Cells. ACS Appl. Mater. Interfaces.

[46] Inaba M., Matsui Y., Saito M., Tasaka A., Fukuta K., Watanabe S., Yanagi H. (2011). Effects of Carbon Dioxide on the Performance of Anion-Exchange Membrane Fuel Cells. Electrochemistry.

[47] Yan J., Hickner M.A. (2010). Anion Exchange Membranes by Bromination of Benzylmethyl-Containing Poly(Sulfone)s. Macromolecules.

[48] Tanaka M., Koike M., Miyatake K., Watanabe M. (2010). Anion Conductive Aromatic Ionomers Containing Fluorenyl Groups. Macromolecules.

[49] Shimada M., Shimada S., Miyake J., Uchida M., Miyatake K. (2015). Anion Conductive Aromatic Polymers Containing Fluorenyl groups: Effect of the Position and Number of Ammonium Groups. J. Polym. Sci. Part A Polym. Chem..

[50] Zhu X., Tokash J., Hong Y., Logan B.E. (2013). Controlling the Occurrence of Power Overshoot by Adapting Microbial Fuel Cells to High Anode Potentials. Bioelectrochemistry.

