# Supplementary material for: Phenolphthalein Anilide Based Poly(Ether Sulfone) Block Copolymers Containing Quaternary Ammonium and Imidazolium Cations: Anion Exchange Membrane Materials for Microbial Fuel Cell
Source: Membranes (Basel). 2021 Jun 20;11(6):454. doi: 10.3390/membranes11060454 (PMC8233788; doi:10.3390/membranes11060454)
Supplement: Supplementary file 1 [file membranes-11-00454-s001.zip › membranes-1249712-supplementary.pdf]

membranes

MDPI

Supplementary Material

Phenolphthalein Anilide Based Poly(Ether Sulfone) Block Copolymers Containing Quaternary Ammonium and Imidazolium Cations: Anion Exchange Membrane Materials for Microbial Fuel Cell

Aruna Kumar Mohanty [1], Young Eun Song [2], Jung Rae Kim [2], Nowon Kim [3,*], Hyun-jong Paik [1,*]

[1] Department of Polymer Science and Engineering, Pusan National University, Busan, 46241, Korea.; akmohanty07@pusan.ac.kr
[2] School of Chemical and Biomolecular Engineering, Pusan National University, Busan, 46241, Korea; duddms37@gmail.com (Y.E.S.); j.kim@pusan.ac.kr (J.R.K.)
[3] Department of Environmental Engineering, Dong-eui University, Busan, 47340, Korea.;
* Correspondence: knwon@deu.ac.kr (N.K.); hpaik@pusan.ac.kr (H-j.P.)

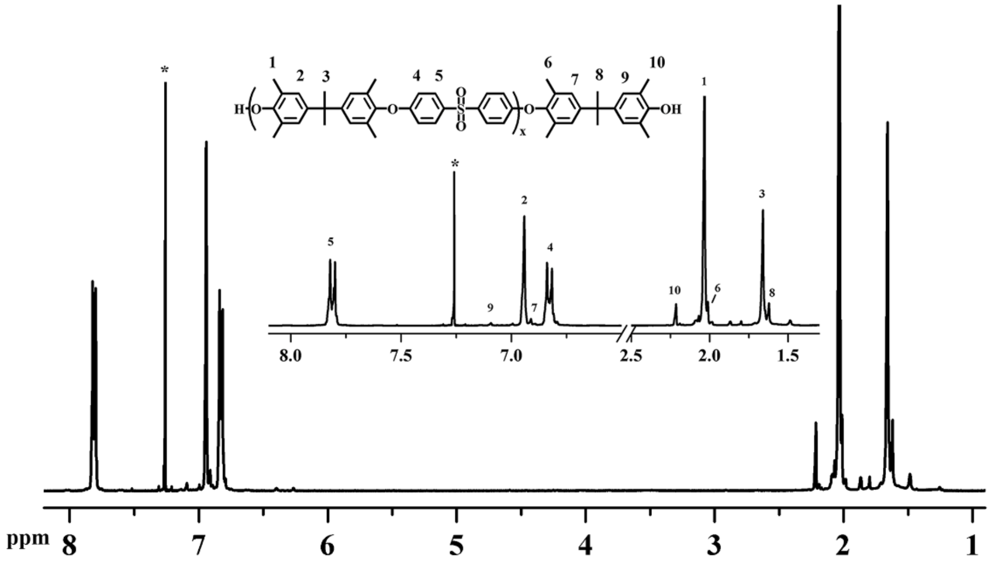

Figure S1. ^{1}H NMR spectrum of PES-1 recorded in $CDCl_3$ at 25 °C.

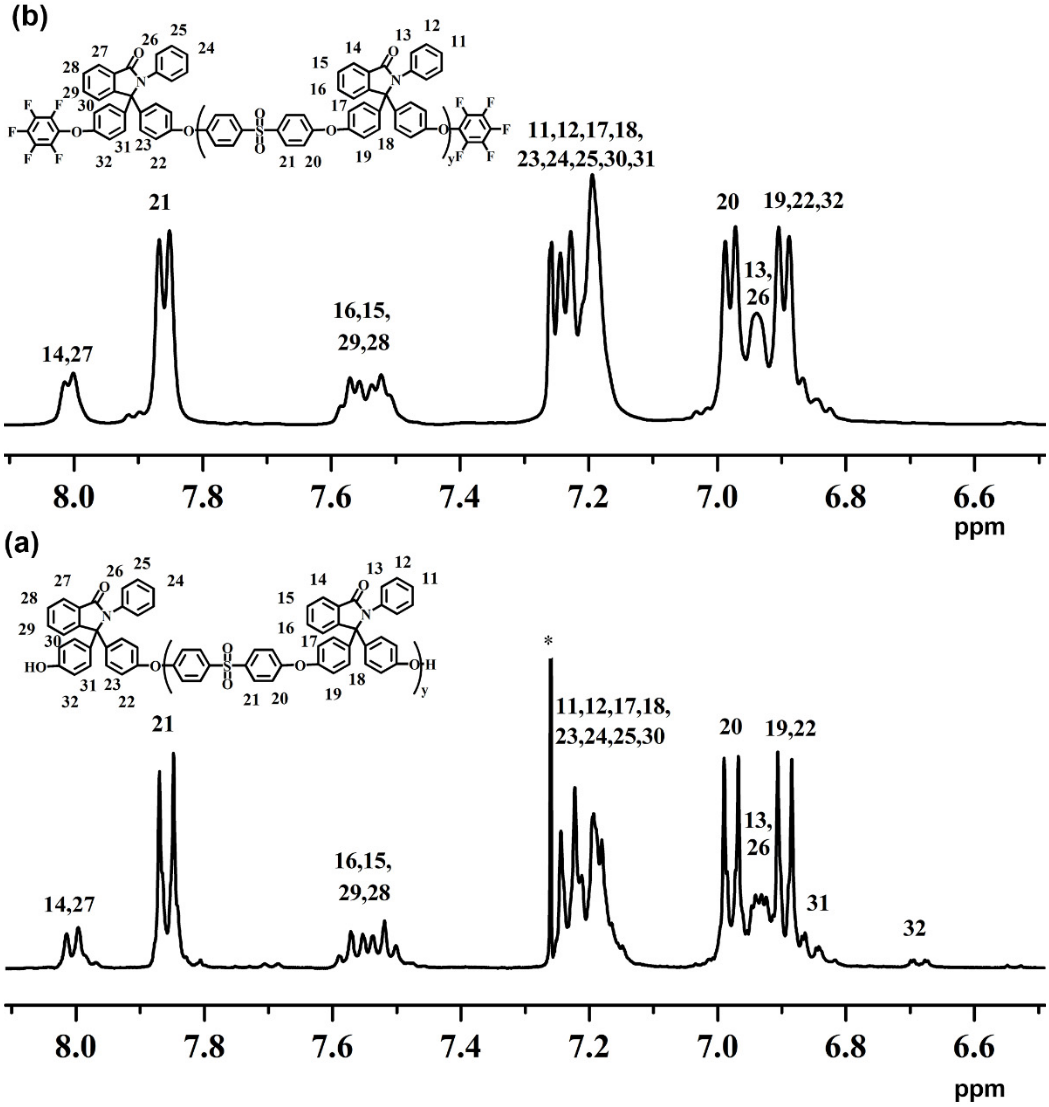

Figure S2. 1H NMR spectra of (**a**) PES-2 and (**b**) PES-3 recorded in CDCl3 at 25 °C.

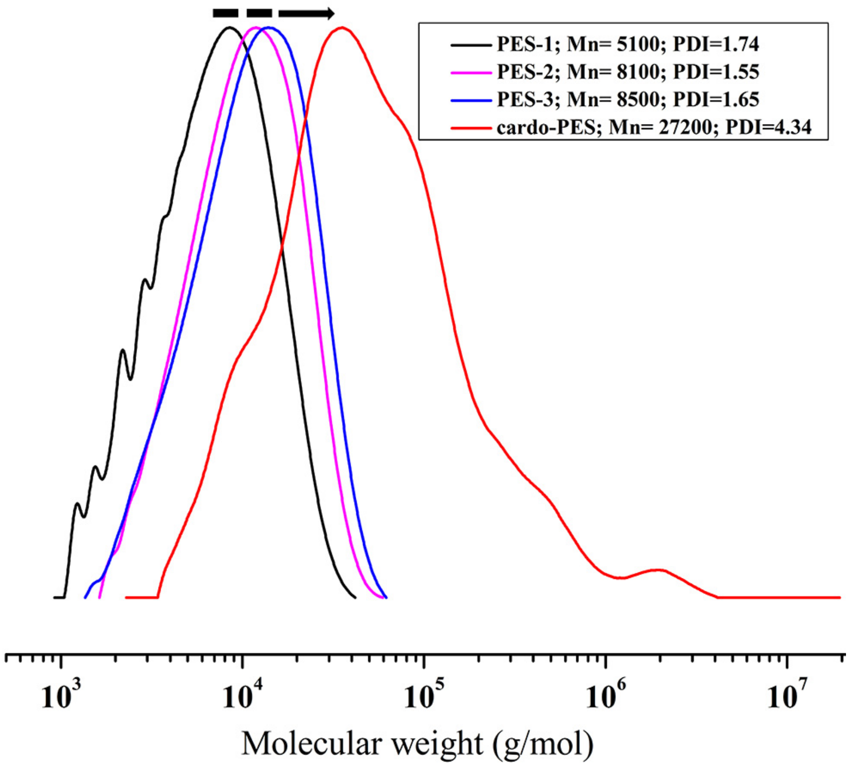

Figure S3. SEC traces of oligomers and precursor block copolymer.

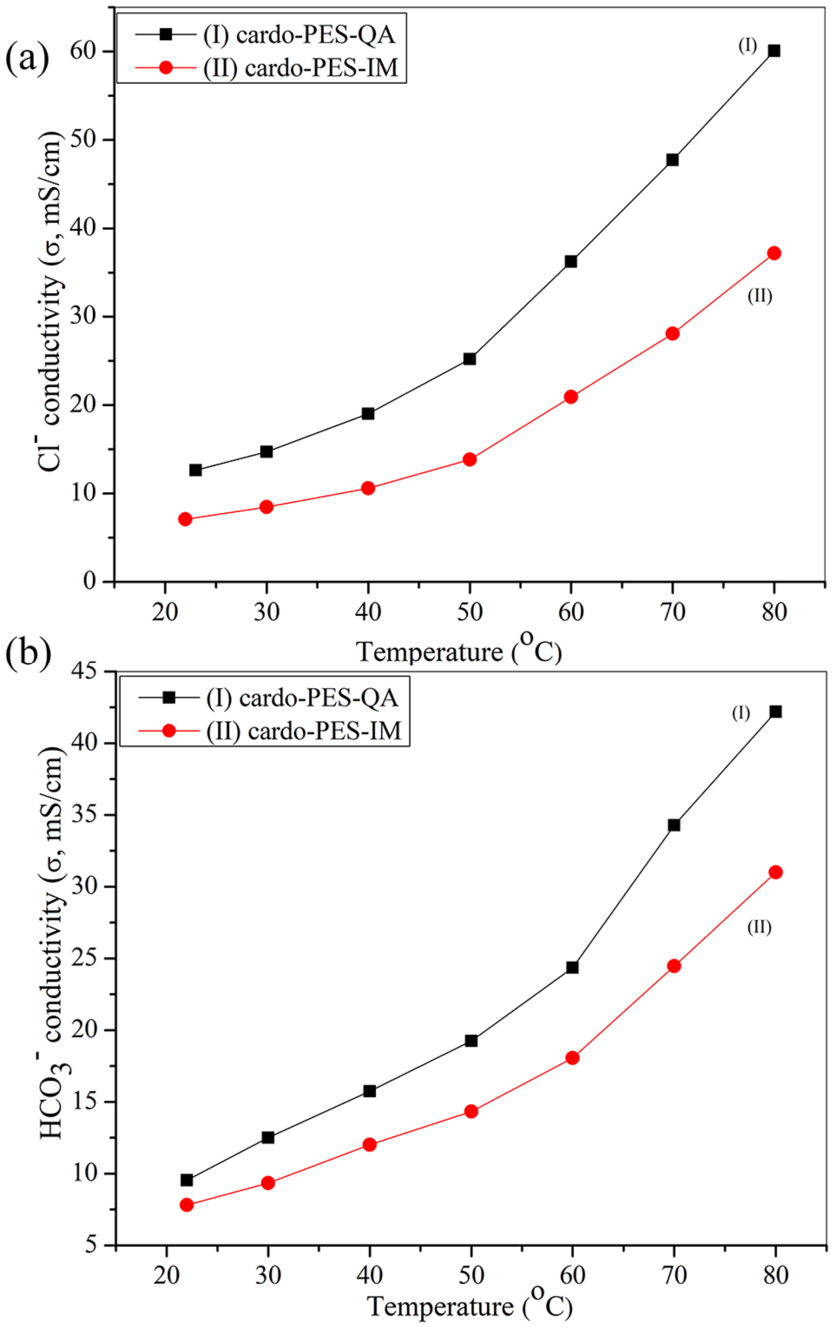

Figure S4. Temperature dependence of (**a**) Cl^{-1} and (**b**) HCO_3^{-1} conductivity of the AEMs.

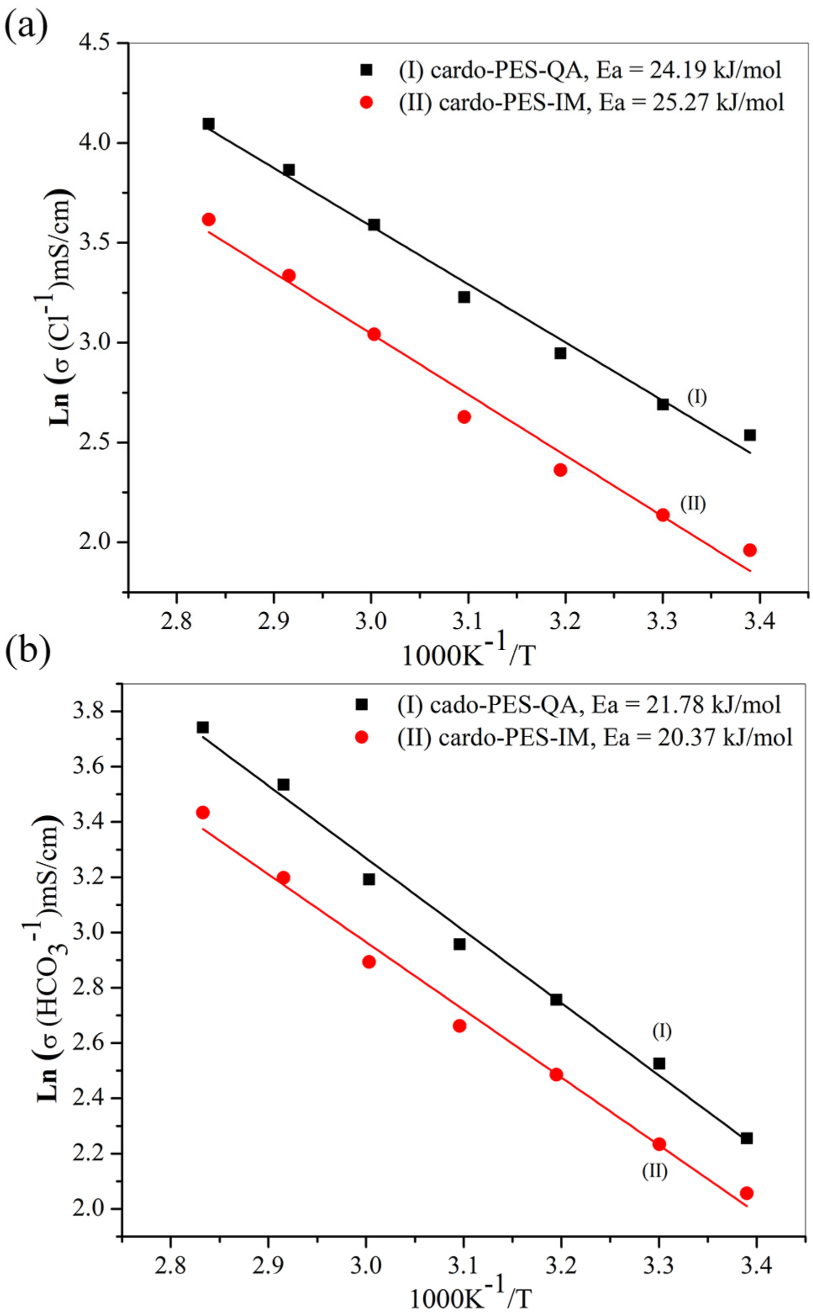

Figure S5. Arrhenius temperature dependence of (**a**) Cl-1 and (**b**) HCO3-1 conductivity (σ) of the AEMs.
